# Human and Non-Human Primate Coexistence in Argentina: Conflicts and Solutions

**DOI:** 10.3390/ani13213331

**Published:** 2023-10-26

**Authors:** Alba García de la Chica, Luciana I. Oklander, Martin M. Kowalewski, Eduardo Fernandez-Duque

**Affiliations:** 1 Instituto de Ecología, Genética y Evolución, Universidad de Buenos Aires, Buenos Aires 1428, Argentina; 2 Owl Monkey Project—Fundación ECO, Formosa 3600, Argentina; 3 Consejo Nacional de Investigaciones Científicas y Técnicas (CONICET), Buenos Aires 1425, Argentina; 4 Instituto de Biología Subtropical (IBS), Consejo Nacional de Investigaciones Científicas y Técnicas (CONICET), Universidad Nacional de Misiones (UNAM), Posadas 3300, Argentina; 5 Neotropical Primate Conservation Argentina, Puerto Iguazú 3370, Argentina; 6 Estación Biológica Corrientes—Centro de Ecología Aplicada del Litoral (CECOAL-CONICET-UNNE), Corrientes 3400, Argentina; 7 Department of Anthropology and School of the Environment, Yale University, New Haven, CT 06511, USA; 8 Facultad de Recursos Naturales, Universidad Nacional de Formosa, Formosa 3600, Argentina

**Keywords:** primates, anthropogenically induced fires, urbanized monkeys, electrocutions, roadkills, infectious diseases, food provisioning, conservation, citizen science, environmental education

## Abstract

**Simple Summary:**

Each of the five primate species inhabiting Argentina faces various threats in terms of conservation that hamper their ability to coexist with human populations. These threats have in common that they are the result of human actions and changes in the landscape, and they all have consequences that may result in human–primate conflict. Furthermore, these changes surely present remarkable challenges for slow-life primates, as they happen too quickly for genetic adaptations to evolve within a timeframe compatible with population viability. Hence, it becomes imperative to delve into the consequences of the different threats that the different species face and the conflicts that are derived from them. We present a detailed compilation of what we consider to be the most relevant and current conflicts between humans and non-human primates in Argentina and describe ongoing national and regional educational, research, and conservation approaches to mitigate those effects.

**Abstract:**

There are five different primate species inhabiting widely distinct ecoregions in Argentina. Each of them faces various threats in terms of conservation and conflicts that hamper their ability to coexist with human populations. We present here some of the drivers known to be the causes of conflicts between humans and primates in the southernmost area of distribution of Latin American primates. We focus our synthesis on two of the biggest sources of conflict: the effects of different anthropogenic disturbances, and human misconceptions concerning the role of primates in the ecosystem. In each section, we briefly characterize the conflicts worldwide and then provide specific cases and examples from Argentina. In the last part of the manuscript, we further describe some ongoing national and regional educational, research, and conservation approaches to mitigate those effects.

## 1. Primate Species in Argentina

**Figure 1:**
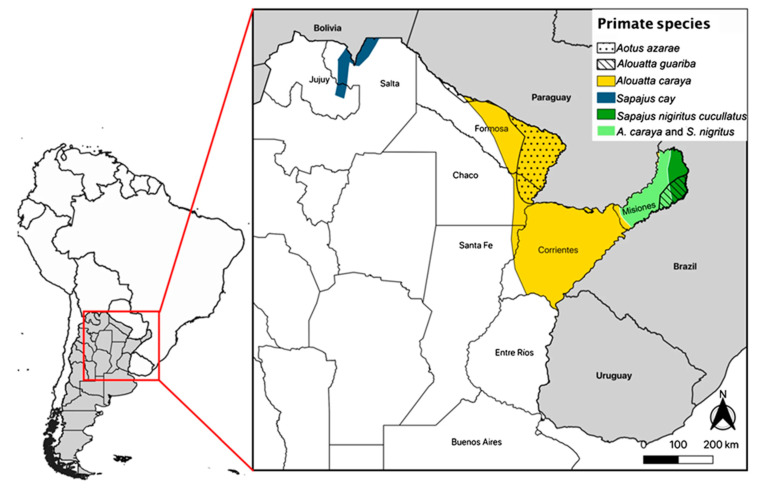
Distribution of all five primate species in Argentina. From [4].

Five non-human primate species (hereafter, primates) inhabit different ecoregions and types of forest throughout northern Argentina. These are two howler monkeys (the black-and-gold howler monkey—*Alouatta caraya*, and the brown howler monkey—*Alouatta guariba clamitans*), Azara’s owl monkey (*Aotus azarae*), and two capuchin monkeys (the black horned capuchin—*Sapajus nigritus cucullatus*, and Azara’s capuchin—*Sapajus cay*) (Figure 1). Even though there are other important differences among them in their biology, behavioral ecology, and social systems, it is beyond the scope of this article to present a detailed description of each species; a good synthesis of the Argentinean primates can be found elsewhere [1,2,3].

Most of these species have their southernmost distribution in Argentina. This, in combination with other factors, such as the fact that for global status, all primate populations with very different ranges of distribution across countries and areas are equally accounted for, may partially explain the different classifications for their level of extinction risk compared to other populations, as recently assessed by Argentinean primatologists (SAyDS, SAREM, 2019) and the International Union for Conservation of Nature and Natural Resources (IUCN) (Table 1). In 2019, as part of the National Primate Conservation Plan, and intending to produce a categorization that considered the particular context of the monkeys in the country, all groups of Argentinean primatologists came together to identify specific conservation threats for all species (see Section 5). In Table 1, we present the conservation threats that were proposed for each species and that were ranked to have a higher impact on the existing populations (for more information: Secretaría de Ambiente y Desarrollo Sustentable de la Nación y Sociedad Argentina para el Estudio de los Mamíferos (eds.) (2019). Categorización 2019 de los mamíferos de Argentina según su riesgo de extinción. Lista Roja de los mamíferos de Argentina. Versión digital: http://cma.sarem.org.ar, accessed on 20 October 2023).

The most important threats for Argentinean primates are forest loss, degradation and fragmentation, illegal hunting, and disease epidemic [5], which are also some of the most frequent threats for primates worldwide [6]. All these threats have in common that they are the result of human actions and changes in the landscape, and they all have consequences that may result in human–primate conflict. Given the relatively slow-life history of primates [7], these changes surely present remarkable challenges for them, as they happen too quickly for genetic adaptations to evolve within a timeframe compatible with population viability. Hence, it becomes imperative to delve into the consequences of the different threats that the different species face and the conflicts that are derived from them. In this synthesis, building on the conservation threats previously identified by experts in Argentina, we present a detailed compilation of the most relevant and current conflicts between humans and primates in the country, while relying on examples from other regions for a more in-depth interpretation.

We reviewed journal articles, book chapters, Argentinean governmental databases, and our unpublished findings documenting, directly or indirectly, contexts of conflict between humans and primates in Argentina. Theses and meeting abstracts were only considered if they worked with primary data collected in Argentina. We used Google Scholar to filter the literature published between 1970 and 2023, in all languages, for all studies containing any combination of three independent search strings of interest in any part of the manuscript (title, abstract, keywords, and body text): Set 1: “primates” AND “Argentina” AND “habitat loss” OR “habitat degradation” OR “landscape modification” OR “fragmentation”; Set 2: “primates” AND “Argentina” AND “pet-trade” OR “mascotism” OR “hunting”; Set 3: “primates” AND “Argentina” AND “roadkills”; and Set 4: “primates” AND “Argentina” AND “diseases” OR “zoonosis” OR “tourism”. For each set, we stopped screening where no new relevant items were found (minimum: 10 pages). To characterize human–primate conflict in Argentina, the focus of this manuscript, we selected all articles (research articles and reviews) that included Argentinean primate populations for any of the topics described. For a more in-depth interpretation, we prioritized those articles that either included the same primate species in other countries (e.g., Paraguay, Brazil) or that carried out their studies in socio-ecological and socio-political contexts more comparable to the ones in Argentina (i.e., studies in/from Latin America).

## 2. Defining Human–Primate Conflict

As interactions between humans and wildlife have increased, the study of human–animal interactions and conflicts have settled into an important, and continuing, area of concern. The importance of the topic is reflected in the existence of relatively new disciplines like Human–Animal Studies (HAS) and Human–Wildlife Conflict (HWC) [8,9,10].

Similarly, the development and growth of ethology and behavioral ecology in the last six decades [11] have started to challenge the notions of non-human animals only as objects or symbols serving functional roles for humans (e.g., food, clothing, company, or prestige). Our improved understanding of animal behavior and ecology moved us to consider the innumerable ways in which our lives intersect with, influence, and are influenced by the lives and deaths of other species [8]. These complex intersections sometimes lead to human–wildlife conflicts, which encompass a wide range of events and have costs for both humans and wildlife [12,13,14]. For humans, some of these costs may include, among others, livestock and crop losses, property damage, or disease transmission [15,16,17]. For animals, at the individual level, they often result in punitive killing; at a population level, they are associated with declines in wildlife populations, threatening the long-term survival of species [18,19,20,21].

Specifically, in the case of humans and primates, the conflict has been considered as “any human–primate interaction which results in negative effects on human social, economic or cultural life, primate social, ecological or cultural life or the conservation of primates and their environment” ([22], p. 1). Nonetheless, in the past, it has been proposed that primates have a more privileged position than other wildlife when conflict arises due to their role as relevant cultural symbols to many human communities and their phylogenetic position as human’s closest relatives [23]. The emergence of ethnoprimatology, the multidisciplinary study of the human/primate interface [24,25,26], has allowed primatologists to include the human dimension and anthropogenic realities as integral factors affecting all aspects of the ecology and behavior of primates [24,27,28,29,30,31]. It follows that careful consideration is required when addressing “conflicts” involving humans and primates to avoid the misrepresentation or exacerbation of existing problems [32,33]. It is then recommended that ethnoprimatological studies are carried out by researchers with experience in the local sociopolitical environment in which they conduct their research [34].

While the combination of human social and cultural variables alongside primate biology can help us better understand the effects of conflict and find sustainable ways for primate–human coexistence [25,30,35,36], there are still many unresolved aspects of human–primate conflict. Indeed, with 60% of primate species now threatened with extinction as a result of human activities [6,37], it is essential to consider the thoughts and actions of humans, within our social and cultural aspects, which ultimately determine the course and resolution of the conflict and the implementation of effective, lasting solutions [25].

Nonetheless, if poverty and income inequality are prevalent in the population, primate conservation or mitigation of conflictive situations will not be a primary social concern. This is somehow paradoxical given the fact that socioeconomic data reveal that, in contrast to developed countries, most nations within primate habitats exhibit elevated poverty rates, significant income disparities, limited human development, inadequate food security, prevalent corruption, and fragile governance structures [38,39]. For instance, in Latin American urban populations, over 25% of city dwellers reside in impoverished settlements, while the wealthiest 20% earn nearly 20 times more than the poorest 20% [40]. Moreover, in some countries, like Argentina, economic progress can sometimes occur at the cost of severe and ongoing environmental degradation [38]. At a more local scale, during the first semester of 2023, the provinces inhabited by primates in Argentina, especially Corrientes and Chaco, were the ones with higher indexes of poverty in the country [41].

Below, we first present some of the drivers known to be the causes of conflicts between humans and primates in Argentina. In each section, we briefly characterize the conflicts worldwide and then provide specific cases and examples from Argentina. We finally describe some ongoing regional, and local educational, research, and conservation approaches to mitigate their effects (Table 2).

## 3. Habitat Loss

Even though primates are particularly susceptible to the negative consequences of habitat loss due to their unique life histories and habitat requirements [42], nearly all primate populations are challenged by some form of anthropogenic disturbance. That disturbance can come from habitat loss and from fragmentation and modified or degraded landscapes [28,43,44,45,46].

Between 2001 and 2018, ca. 30% of forest canopy cover worldwide was lost due to human activities, almost half of it in the Neotropics (i.e., Mexico and Central and South America) [38]. A considerable part of this loss comes as a consequence of agricultural expansion, which, in Chaco’s forests, was translated into an overall loss of 22.5% of land between 2000 and 2010 [47]. Indeed, at a global scale, the Dry Chaco had the highest deforestation rates for tropical forests during the period 2000–2012 [48], and between 2000 and 2019, the overall extent of Dry Chaco forest cover loss was estimated at 9.5 million ha. [49].

Concern about protecting remaining native forests in the Argentine Chaco led to a national Forest Law, passed in 2007 (Ley de Presupuestos Mínimos de Protección Ambiental de los Bosques Nativos 26.331). As part of the implementation of the law, the Ministry of Environment and Sustainable Development annually reports the results of the monitoring of native forests with the goal of detection and quantification of processes of natural or anthropic origin that modify the structure and extension of natural forest ecosystems. The latest report, published in 2021, identified a loss of 158,746 ha. The provinces inhabited by primates were among the ones most affected by forest loss, especially Formosa and Chaco with 30,138 and 21,460 ha of forest loss, respectively. It is worth noting that 55% of the deforested land in 2021 was illegally altered on “protected” areas declared of high conservation value [50].

Notwithstanding the type and causes of habitat loss, these are highly variable and can have long-lasting effects across all aspects of primate ecology and behavior [51]. For instance, fragmentation can impact primates’ home range, activity patterns, social interactions, group composition, or even social and mating system changes due to scarce or insufficient genetic flow [34,42]. It can further lead to dietary adjustments, such as the incorporation of human foods including crops and provisioned items [34].

It follows that understanding how the different habitats are affected by forest loss and landscape modification is critical to understanding the effects of these changes on the different species´ ecology and behavior.

For example, likely important for howler monkeys in Argentina was the loss of 46,990 hectares of native Paranaense forest, and 2,773,607 hectares of native areas in the Parque Chaqueño region during the period of 2007–2017 [52]. However, when comparing howler monkeys to other species in Argentina, it becomes apparent that they tend to occupy relatively smaller forest fragments. In Brazil, the occupancy of *A. caraya* is influenced by various factors, including the shape of the local patches, the amount of forest present, and the level of fragmentation in the landscape [53]. A study conducted across the *Alouatta* genus ranging from Mexico to Argentina, suggested that these monkeys exhibit the highest occupancy rates even within small forest patches [54]. Studies of howler monkeys in Argentina showed that neither fecal cortisol metabolite levels nor feeding patterns and diet composition were remarkably affected by fragmentation or fragment size, respectively ([55,56] but see: [49,57]). Nevertheless, individual howler monkeys’ capacity to survive in these poor-quality, fragmented patches does not ensure the sustained survival of populations in such conditions [51]. For instance, the loss of their natural habitat and the subsequent fragmentation severely restrict the howler monkeys’ ability to disperse and diminish gene flow within Argentinean populations [58].

The reduced connectivity of forested areas along river corridors could also lead to the isolation and subsequent loss of genetic diversity among owl monkeys (*A. azarae*) in Formosa [1,59,60]. Since 1990, the replacement of native forests with large-scale monocultures has been particularly intensified in northern Argentina and southern Paraguay [61], where *A. azarae* populations live. Previous research on naturally fragmented forest patches in Guaycolec Ranch, Formosa, has shown that groups inhabiting these isolated habitats were, on average, smaller than groups in continuous gallery forests [60]. For species inhabiting Misiones and the Paranense rainforest, such as *A. caraya*, *A. guariba*, and *S. nigritus*, a process of Atlantic Forest replacement by forest plantations, agricultural crops, and livestock was documented in Misiones between 1973 and 2006 (Izquierdo et al. 2008). In contrast to howler monkeys, *S. nigritus* have relatively large home ranges in Argentina (range: 81–293 ha) [62] and do not inhabit small patches [63]. Hence, the impact of fragmentation on this species could, potentially, be more severe than the others.

Lastly, with regard to habitat loss in the Yungas, the only region where *S. cay* is found in Argentina, it is worth noting that between 2007 and 2016, a total of 67,578 hectares of forest were lost in the Yungas [52]. Forest extraction in the Yungas is extensive and occurs on a considerable scale, both legally and illegally. Over 90% of the original Pedemontana Forest, located on deep soils, vanished when it was converted into extensive sugarcane crops between the 1930s and 1950s, and more recently, into soybean plantations [64]. This reduction in forested areas particularly impacts *S. cay*. During the winter season, when environmental conditions are unfavorable in the higher Yungas areas (low temperatures and scarcity of food resources), they descend from the mountains to the Pedemontana Forest [64].

Studies have shown that forest loss between 2000 and 2019 has altered landscape connectivity for the Dry Chaco’s associated biota across Argentina, Bolivia, and Paraguay. The presence of numerous forest fragments and the distances between them indicate that some of the mammals characteristic of this biome may experience adverse effects from this fragmentation in the coming years [49]. Particularly in Argentina, due to the growing global demand for soybeans and ineffective implementation and enforcement of forest conservation laws, modeling approaches suggest that both the forest area and connectivity in the region are projected to drastically decline [47].

### 3.1. Anthropogenically Induced Fires

One of the most noteworthy forms of anthropogenic disturbance in Argentina is the impact of intentional fires (Table 2). While some fires occur naturally, in many rural areas in the Provinces of Corrientes, Formosa, and Misiones in northern Argentina, there are still customary harmful cultural practices that involve the use of fire for grassland management and clearance [65,66]. Additionally, because of climatic events associated with drought conditions, such as those brought on by La Niña events. the course and intensity of these uncontrolled burns often become unpredictable and extremely dangerous.

The destructive power of fire in the area was made evident recently. In 2020, ten consecutive months of unusually low amounts of rainfall facilitated the conditions for the formation and maintenance of fire spotlights [67]. That year, all but one of the 23 provinces in Argentina registered fires (MAyDS, 2020), and more than half of the loss of native forests during that period (54% of 333,000 ha) was caused by fires. Between August 2022 and July 2023, there were 20,165 high-confidence fire alerts in the country (GlobalForestWatch, 2023).

**Figure 2:**
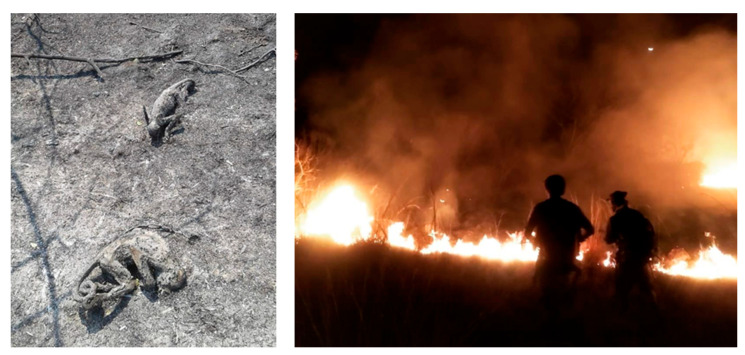
Images of the EBCO field site after the anthropogenically induced fires of August–October 2020 show two calcined howler monkeys (**left**), and firefighters controlling an active line of fire (**right**). Photo credit: Marita Romero and Adriana Vallejos.

In terms of subsequent conflict and how these fires affected primates, the most alarming example is the one experienced at the Estación Biológica de Corrientes (EBCO). During August and October 2020, this multi-decade study site suffered two consecutive intentional fires (to obtain new pasture for cattle) that burnt at least half of the area where researchers had monitored black-and-gold howlers for almost 40 years [68]. The field site lost half of the surveyed groups (an estimated 10 groups—80 individuals—disappeared during the fires or the subsequent months) (Figure 2). The remaining, surviving groups are still located and overpopulating the forest patches that the fires did not reach. Ongoing research is focused on assessing the potential multi-year/multi-generation effects of fires on the survival of the population.

**Figure 3:**
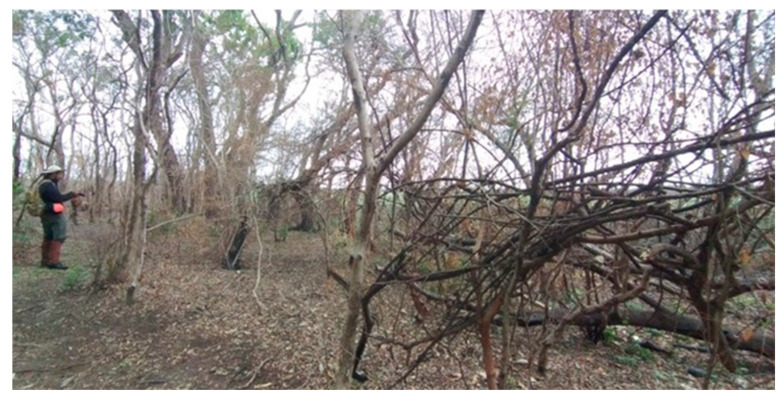
National ranger collecting data on one burned patch of forest at the Iberá National Park, Corrientes. Photo credit: L. Oklander.

Another example comes from the Cambyretá portal of Iberá National Park in the province of Corrientes. In 2022, led by Dr. L. Oklander, researchers and park rangers collected data on 29 fire-affected forest patches to assess damage to howler monkey populations (Figure 3). The evaluation included data on the overall health of monkeys, available resources, accessibility to nearby patches, and the potential restoration of the landscape corridor. Monkeys were found in 7 out of the 29 forest patches. Most groups were in relatively good health. Still, one group, located in a particular low-resource area, required specialist-supported food supplementation (Oklander, unpublished data).

Lastly, the Montane Forest in the Yungas region of Argentina faces further threats from logging and fires used to clear pasture for cattle [69]. These induced fires occur more frequently during the driest period of the year, between August and November. In 2013, another particularly dry year, nearly 200,000 hectares of Southern Yungas were consumed by fires [70]. Unfortunately, there are currently no available data on the effects of fires on the *S. cay* of the Yungas.

### 3.2. “Urbanized” Monkeys

Human–primate conflict is not limited to rural areas. As human populations expand, conflict with primates is now frequent in a variety of settings including urban and suburban areas [22].

While some primates adjust to increasingly urbanized new habitats, others do not [71,72,73]. Urbanized areas contain a variety of human-made structures that may limit primate movements and threaten their lives [74]. For instance, they often suffer serious physical injuries or die because of collisions with vehicles, electrocutions in power lines, and/or domestic dog attacks [71,75,76,77,78].

In regions of Argentina, Brazil, and Paraguay, individuals and/or groups of howler monkeys (*A. caraya*) range in small, forested areas of university campuses and parks [79,80,81]. They are also found in the middle of cities, like the capitals of Corrientes, Chaco, Misiones, and Formosa provinces [82], where they regularly use power cables to move (see a more detailed description of the effects of power lines below). In the provinces of Misiones and Formosa, L. Oklander and A. Garcia de la Chica have participated in the capture and relocation of howler monkeys that were ranging in the cities of Posadas and Formosa, respectively.

Living in urban areas has effects on the survival of monkeys, as well as on their behavior and physiology. For example, when comparing the glucocorticoid levels (GCC) of male and female black-and-gold howler monkeys (*A. caraya*) in urban and rural areas in the province of Corrientes, those living in urban areas had lower GCC levels than the ones in rural zones. The limitations of hormonal evaluations from feces notwithstanding [83,84], this difference could be indicative of a different physiological state in the two habitats [81]. In the city of Pilar, in southwest Paraguay, howler monkeys living in urbanized areas showed similar activity budgets to those living in natural environments, which could suggest a relatively good adaptation to increased urbanization [85]. Still, monkeys in this city seemed to prefer spending more time in areas with lower levels of anthropogenic risk (e.g., barbed or electric wires, roads) [86]. Yet, while many aspects of the monkeys´ ecology and behavior may be conservative, like their movement patterns or daily ranges, other behaviors may show variation in response to changes in the environment or abiotic factors that are a product of anthropogenic habitats [87]. The multi-year project initiated by the EBCo´s group suggests that urban groups in the cities of Corrientes and Resistencia may be genetically and spatially isolated; since very rarely have individuals been observed to successfully disperse, the risk of endogamy is potentially high (Kowalewski, unpublished data).

Wild Azara´s owl monkeys inhabit peri-urban areas along the San Hilario River in the city of Formosa (García de la Chica, *pers. obs.*). Notwithstanding the differences between the cathemeral owl monkeys in Argentina and the nocturnal owl monkeys in the tropics [88], a study of Colombian night monkeys (*Aotus lemurinus*) in peri-urban forest in the city of Manizales suggested sufficient behavioral and dietary flexibility in exploiting the resources available in urban areas [89].

This presence of monkeys in urban areas generates a series of conflicts. Thus, a critical aspect of the survival of monkeys in urban environments is how they are perceived by people. For example, in Argentina, it is often the case that citizens either directly interact with the monkeys by touching and providing them with food that is not good for them (i.e., bread, candy, and fruits and vegetables not part of their diet); or ask the local authorities to remove them, given the discomfort that their excrements and/or loud vocalizations cause (Garcia de la Chica, *pers. obs*). In the city of Pilar, Paraguay, people’s attitudes toward the presence of howler monkeys (*A. caraya*) were highly positive; instead of perceiving them as problem-makers, the majority of interviewees believed that they brought benefits to the area and that they should be protected from potential risks in the urban environment [80]. Even though similar studies are still needed in Argentina, the findings from this small community with many cultural similarities to the nearby Argentinean region are promising; these hopeful results have the potential to provide the basis for collaborative, community-based, conservation plans that will find solutions to “urbanized” monkeys. The EBCo group has developed and implemented several citizen science and environmental education projects with the local community of the city of Corrientes (see more details below). The main goal of these projects was to promote the long-term establishment of new urban ecosystems while turning cities into more nature-inclusive environments [90].

Despite the lack of studies specifically addressing the progression of urbanization effects on primates in Argentina, abundant literature suggests that the situation has worsened. Already in 2011, modeling approaches showed that considering migration dynamics and demographic growth of human populations, in all future scenarios, it is expected that native Atlantic Forests will decrease by 18% to 39% of their current coverage by 2030 [91]. Furthermore, the impact of urbanization on biodiversity may pose major challenges in Latin America, given that many cities are situated in or near regions characterized by high species richness and endemism. For instance, in Argentina, rich coastal ecosystems and river deltas were classically the centers for population settlements and urban growth [92]

### 3.3. Power Lines and Electrocutions

Human-made structures, such as high-voltage power lines, can be deadly to primates [93,94,95,96]. This is particularly true for large arboreal species (≥8 kgs) that can easily access them [97]. Power lines’ negative effects can range from serious injuries to immediate death by electrocution [98,99]. Several studies of primates have reported that 30–40% of electric shocks result in death (*Macaca mulatta*: [99]; *Alouata guariba clamitans*: [74]; *Semnopithecus vetulus vetulus*: [100]).

**Figure 4:**
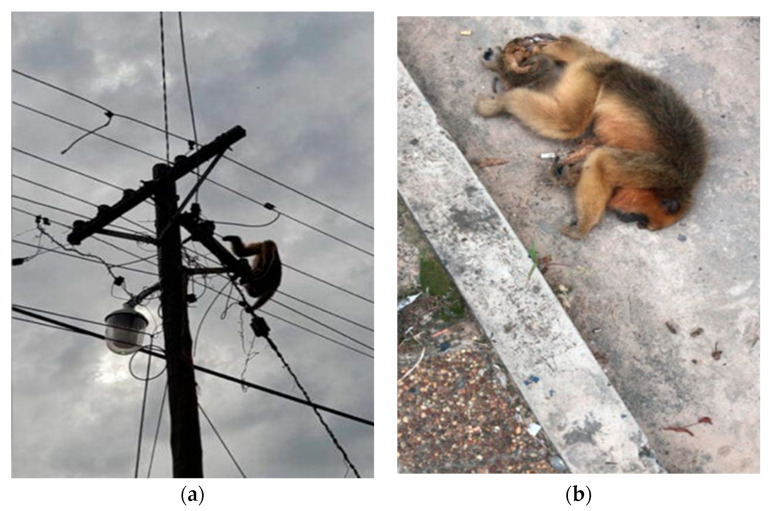
Lethal electrocutions of howler monkeys (*A. caraya*) in (**a**) the city of Posadas—Misiones province and (**b**) the city of Formosa—Formosa province. Photo credit: (**a**) Misiones Online (www.misionesonline.net/2022/07/14/mono-electrocutado-en-posadas, accessed on 20 October 2023); (**b**) La Mañana Online (www.xn--lamaanaonline-lkb.com.ar/noticia/48669/un-mono-caray-provoc-un-cortocircuito-en-el-tendido-elctrico-y-dej-sin-luz-al-centro-de-la-ciudad, accessed on 16 September 2023).

In Argentina, deadly cases of electrocution are becoming more notable as they are portrayed in the news and media. Deadly cases of electrocution have been reported for howler monkeys, the largest primate species in the country, in four of the five provinces where they are found (examples of two provinces in Figure 4). In the province of Corrientes, from January to September 2023, there were 20 cases of lethal electrocutions of howler monkeys (Estación Biológica Corrientes/Kowalewski, *per. obs*). In the city of Iguazú, in Misiones Province, between 2020 and October 2023, five electrocuted monkeys (three *S. nigritus* and two *A. caraya*) were received at the Güira Oga rescue center, of which two died due to serious injuries and three were satisfactorily rehabilitated (Di Nucci, *pers. comm.*).

It is worth noting that the use of power lines by primates, and subsequent accidents, are not limited to urbanized areas. They also occur in peri-urban environments, where lethal electrocutions of howler monkeys occur when they use power lines to move across fragmented patches of forest [76]. To mitigate the negative effects of interactions between primates and human-made infrastructure, it is possible to build structures specifically designed for wildlife use. Building bridges for wildlife can have multiple positive effects; it may help connect fragmented habitats, reduce disease transmission from wild to domestic animals, and reduce predation events by dogs [74,76,77,79,101,102,103,104].

### 3.4. Roadkills

**Figure 5:**
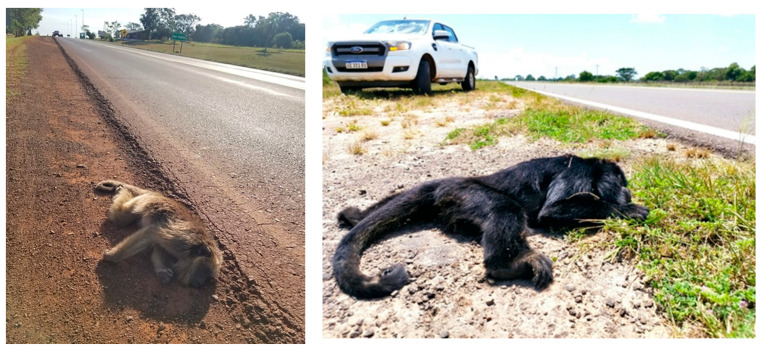
Images of roadkills in Argentina show two dead howler monkeys, an adult female in the locality of San Carlos, in the limit between Corrientes and Misiones provinces (**left**), and an adult male in the locality of Itatí, in the province of Corrientes (**right**). Photo credit: L. Oklander.

Another cause of conflict between humans and primates is the rapid growth of road networks worldwide, which, particularly in tropical and subtropical regions, is increasing the threat of vehicular collisions with primates [105,106,107]. Intending to store and organize information on these incidents in a standardized dataset, a group of researchers recently developed the Global Primate Roadkill Database (GPRD) [75]. This open-access online repository has documented over 2800 roadkill incidents from 1987 to February 2023, involving at least 107 primate species from 41 countries, including Argentina. Specifically in Argentina, a similar initiative was recently created (2020) to promote the participative monitoring and registering of wildlife roadkills (Figure 5).

The *Red Argentina de Monitoreo de Fauna Atropellada* (RAMFA) systematizes the data collected by citizens through a cellphone app. As of April 2023, there were 13 recorded cases of deadly roadkills (10 *A. caraya* and 3 *S. nigritus*) in the provinces of Chaco, Corrientes, and Misiones (www.fauna-atropellada.org.ar, accessed on 20 October 2023). While the number of primate deaths recorded is low compared to the overall number of wildlife roadkills (5846 cases), the initiative has the potential to be useful for future scientific analysis and to be used by Argentinean conservation practitioners and policymakers.

### 3.5. Crop Foraging

In this section, following what other authors have proposed, we choose the term crop foraging over crop raiding. The latter term may imply, and reinforce, the idea of conflict and, more importantly, the idea of perpetrators and victims in such conflict [108]. Instead, crop foraging simply refers to the addition of agricultural foods to the diet of primates. Crop foraging is then another cause of conflict between humans and primates when it is perceived as an unaffordable economic cost or when the cost has been imposed externally. It is a cause that increases the probability of retaliatory killing of some species [22,108,109].

Due to the expansion of agriculture and subsequent habitat loss and landscape change, humans and primates come in conflict as the latter regularly feed on crops or bark-strip in plantations of trees of commercial value [108,110]. A total of 57 primate taxa, present in 38 types of agroecosystems worldwide, have been involved in this type of conflict [6]. In Brazil, brown howlers (*A. guariba clamitans*) exploited six different cultivated fruit or seed species sources [111], and capuchin monkeys (*Sapajus* spp.) are well known for the consumption of corn, sugarcane, and manioc [112,113].

It is the case that species that live in larger social groups, with more flexible or omnivorous feeding ecologies, higher levels of semiterrestrial locomotion, and bold temperaments, are more likely to forage on crops than more specialist species [72,108]. In Argentina, the only species reported to engage in crop, or bark-stripping of pines is the black-horned capuchin monkey (*Sapajus spp.*). Because of frequent conflicts with forestry companies, the species is particularly exposed to retaliatory killing [63,110]. A study in the Atlantic Forest of eastern Paraguay, registered two groups of *S. cay* eating seeds from pines of the Slash Pine (*Pinus elliotti*) in a human-made plantation bordering the forest, although retaliatory killing or other contexts of conflict due to crop foraging were not mentioned in the study [114]. Neither howler nor owl monkeys are known for this behavior in Argentina, and we lack information on conflicts due to crop foraging for *A. azarae and A. caraya.* Nonetheless, in the province of Corrientes, there have been recent reports of howler monkeys eating fruiting trees from small farms that use this production for homemade jams sold at local markets, initiating a new kind of conflict between producers and howlers (Kowalewski, *pers. obs*). In Colombia, nocturnal wild owl monkeys (*A. lemurinus*) inhabiting peri-urban areas consume cultivated avocados (*Persea Americana*) and a species of banana (*Musa × paradisiaca*) [89]. It is possible that especially in peri-urban areas with easier access to plantations, howler and owl monkeys show higher dietary flexibility and take advantage of these foods, including them in their diet.

## 4. Human Misconceptions about Primate Biology and Behavioral Ecology

### 4.1. Transmission of Infectious Diseases

Argentina has reported the presence of several, medically important, mosquito-borne flaviviruses, including Dengue virus (DENV), St. Louis encephalitis virus (SLEV), West Nile virus (WNV), and Yellow Fever virus (YFV), all of them linked to human diseases [115]. It follows that closer proximity and contact with primates can trigger opportunities for zoonosis and epidemics [6,116]. While this is undoubtedly true, there are some cases where unjustified fears of the risk of contagious disease have created conflicts that jeopardized the lives of primates [117,118]. An example of this has occurred in Argentina with the outbreaks of yellow fever.

In Argentina, the infectious disease that has affected primates the most is Yellow Fever virus. Over the years, yellow fever has caused severe illness and the death of hundreds of howler monkeys (*Alouatta* spp.) in the country [119,120]. The first reported cases of dead howler monkeys occurred in the province of Misiones in the 1960s; at the same time, yellow fever cases were reported in humans [121]. In 2001, 80 howlers died of yellow fever near the Brazil–Argentina border, and between November 2007 and October 2008, yellow fever decimated the already small remnant population of brown howlers and black-and-gold howler monkeys of the Atlantic Forest of Misiones province [2,122]. In southern Brazil, the same outbreak decimated many howler populations, and a more recent one, in 2017, caused thousands of deaths [123]. It follows that the genus *Alouatta* is exceptionally susceptible to lethal infections of Yellow Fever virus. Expectedly, the lethal effects of the virus can be even more prominent in those populations with a reduced effective size due to limited genetic flow, such as the case of the subpopulation that inhabits the Atlantic Forest at the border between Brazil and Argentina in Misiones province [120]. While the appalling effects of yellow fever are certain [123], it is worth noting that howler monkey populations of the humid Chaco (Corrientes, Chaco, and Formosa provinces) registered substantially fewer deaths during the last two yellow fever outbreaks (2008/2017). It is the monkeys´ susceptibility that makes them of high epidemiological importance, major indicators of enzootic disease outbreaks in forest areas, and health sentinels for prompt detection of the virus [4,116,120]. However, for this same reason, mostly in rural areas, people sometimes are of the wrong impression that howler monkeys can transmit the disease to humans, which can lead to the killing of the monkeys when encountered. Of course, the only way to attenuate the outbreaks is to ensure human vaccination against the virus, combined with information campaigns and education programs and protection of priority areas for monkeys´ conservation [4] (see next sections).

A very similar situation took place in 2022 with the outbreak of “monkeypox”. After an increase in attacks against primates because people considered them a potential source of infection for this disease [117], the World Health Organization, rightly and officially changed the name of the virus to Mpox. Most attacks happened in Brazil, the world´s second most affected country [124]. In Argentina, we lack data on official or published reports.

### 4.2. Food Provisioning

Intentional or negligent food provisioning can generate conflicts between humans and primates since it can increase the likelihood of attacks and opportunities for disease transmission [73,125]. It can further have negative consequences for primates such as changes in individual activity and ranging patterns [126], or an increase in intra-group aggression levels [127].

Food provisioning is a common problem in areas where primates and humans co-occur (e.g., urban areas), but mostly in, or near, tourist areas. Several studies have assessed the impact of tourism or provisioning on primates worldwide [128,129,130], showing that sociocultural elements (e.g., age, gender, education, and religious beliefs) explain why humans engage in these behaviors at tourist sites, such as [128,131]. Overall, humans feel compelled to give primates food because of the desire to observe them closely and concerns over decreasing food resources for wildlife.

**Figure 6:**
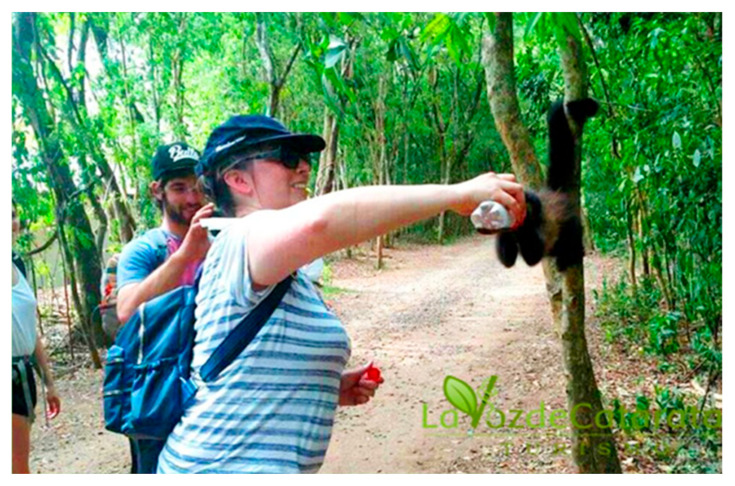
Tourist feeds a capuchin monkey (*S. nigritus*) at the Iguazú National Park. Photo credit: lavozdecataratas.

In Argentina, the problem and conflicts associated with food provisioning are of particular importance at the most visited National Park of Argentina, the Iguazú National Park. This site is the habitat of capuchin monkeys (*S. nigritus*), a species that has the propensity to interact with humans given their omnivorous feeding ecology, foraging strategies, small body size, and terrestrial habits [132]. Preliminary studies have not found a direct association between behaviors associated with anxiety and the context of direct interactions with tourists, nor have they found an increased risk of parasite and pathogen infection in contexts of mass tourism [133,134]. Nonetheless, researchers registered more than 2000 direct interactions between humans and capuchin monkeys in a six-month period, and a higher frequency of food provisioning during periods of food scarcity [131]. Since the year 2000, there has been a progressive increase in national and international tourism at the Iguazú National Park, a situation that has worsened the now pervasive problem of food provisioning there (Figure 6).

Besides the obvious negative consequences for the monkeys, conflicts are frequent in the park, particularly with the group whose territory is in the area where a Sheraton Hotel is located. The problems with this hotel date back to 1999, and despite numerous warnings and notices, the tourists staying there regularly feed the monkeys. This has resulted in monkeys climbing to the balconies of the hotel, and/or opening sliding windows to enter rooms and take food or belongings from tourists. Fortunately, and different from the consequences of conflict with another mammal in the area, the coatí (*Nasua nasua*), in the case of capuchins, no serious injuries, such as bites, have been officially registered as of September 2023. Still, the consequences of these could be much more dangerous.

Although there are informative posters and signals about the negative effects and dangers of feeding the animals all along the trails in the park, these do not seem sufficient to reduce the interactions with the monkeys and mitigate conflicts.

### 4.3. Illegal Pet Trade

Hunting pressure further influences the persistence of primate species [54,135]. While specific data on hunting pressure and illegal pet trade for monkeys in Argentina are limited, information from primate populations in other parts of Latin America provides valuable insights into this context of conflict. For instance, since 1974, an average of 1100 wild-caught live owl monkeys were traded per decade with CITES registration, primarily from Peru and Brazil to the United States for biomedical research [136]. Despite national bans on primate exports and the initiation of captive breeding programs, illegal trafficking of *Aotus* monkeys for biomedical research continues, primarily in the tri-border area of Brazil, Colombia, and Peru [136,137].

Regarding the pet trade, there has been limited research and public attention focused on the trafficking of wild primates in South America, with data frequently derived from unpublished reports provided by national authorities. In the case of *Aotus* spp., extrapolated data from wildlife trafficking hotspots and confiscations suggest the underreporting of owl monkey trafficking [136,138].

In Argentina, owl monkeys are occasionally trapped for pet trading due to their small size, appearance, and docile nature ([139], Dirección Nacional de Biodiversidad, unpublished data). However, their nocturnal and cathemeral habits modulate their interactions with humans, potentially limiting their popularity as pets compared to other monkey species [140]. Indeed, in Argentina, as in other parts of Latin America, *A. caraya* is the species that faces major capture pressures for illegal pet trading, as evidenced by the frequent encounters during law enforcement operations ([139], Dirección Nacional de Biodiversidad, unpublished data) and their presence in rescue centers (see below). In Misiones, cases of illegal pet trade of *S. nigritus* have also been reported (Dirección Nacional de Biodiversidad, unpublished data).

### 4.4. “Rescue” Centers

As previously stated, howler monkeys (*A. caraya*) were the mammal species most often confiscated by national and local authorities, or voluntarily surrendered to state-managed animal rescue centers, during the last decade. This is explained by the more frequent presence of the species in urbanized areas, and because it is the species most frequently hunted for illegal pet trade (Dirección de Inspecciones, Brigada de Control Ambiental, Ministerio de Ambiente y Desarrollo Sostenible, Argentina). The voluntary surrender of howlers occurs due to the inconvenience caused by keeping a wild animal as a pet when owners are no longer able, or willing, to care for the monkeys or upon learning that their possession is illegal. It is also the case that local authorities often capture howler monkeys in urbanized areas following complaints from citizens due to unmitigated conflict.

When these situations happen, confiscated and captured animals ultimately arrive at rehabilitation centers or sanctuaries [141]. Despite the lack of financial support for state rescue centers, the number of individuals kept in ex situ settings is sharply increasing, which makes it very difficult to sustain these primates in captivity. In Latin America, including Argentina, rescue centers are frequently overcrowded and rarely meet basic welfare conditions [142]. Altogether, the unsustainable situation at these centers results in many cases of animals being released back into natural environments. These releases are frequently lacking in adequate prior considerations. The animals may be released in natural environments different from the original ones where they were captured, and releases most likely occur without any genetic analysis profiling, behavioral reeducation, or complete health screening and post-release monitoring of individuals to evaluate success. In general, this process of returning confiscated animals to the wild is referred to as liberation, release, or translocation. However, people generally use the term “reintroduction”, which should be reserved for cases when animals are returned to areas within the species’ original distribution where populations are now extirpated. In Argentina, releases are frequently undertaken in forest areas near the rescue centers, where other healthy monkey populations exist.

Despite the strong support that these releases have from the media and public [6,143], they may likely be detrimental to the animal and/or the environment if decisions on where to release are not based on solid scientific evidence. One important consideration in planning a release is to conduct genetic evaluations to avoid the admixture of distinct evolutionary lineages. An evaluation of the genetic cluster of origin of 17 translocated howler monkeys in Argentina showed that only four of them had been released in sites corresponding to their cluster of origin [144]. Unfortunately, it is not usually the case that releases are followed up and that information and data on negative outcomes are published. In Argentina, the only published record of a reintroduction comes from the province of Misiones, where a private rescue center (Güirá-Oga) started a trial reintroduction of 12 howler monkeys (*A. caraya)* into a protected area on a 160 ha island in 2017. Animals were released after they had undergone a health screening, behavioral rehabilitation, and genetic analysis to confirm their origin [120]. Five months after the release, genetic and observational data showed that two of the monkeys had died due to natural predation events, two had disappeared, and, as of August 2023, eight individuals were still alive and in adequate health conditions [145].

Nonetheless, the lack of sufficient government resources to handle confiscated animals has led to the establishment of various other private centers that frequently conduct inappropriate behavioral rehabilitation procedures. Ignoring scientifically validated rehabilitation procedures [146], in some of these centers, monkeys are taken care of with extended close contact with humans, including tourists. An additional problem is created when some of these centers, which are usually not located within the natural distribution area of the monkeys, allow the unplanned reproduction of individuals. This creates a scenario where captive populations, not suitable for reintroduction, keep increasing each year due to uncontrolled reproduction. Finally, in Misiones, the possible release of individuals from captivity, mostly from another congeneric species (*S. cay*), could potentially lead to hybridization and, consequently, decrease the population viability of the native species, *S. nigritus* [147]

## 5. Regional and Local Approaches to Mitigate Human–Primate Conflict in Argentina

To enable human–primate coexistence without conflict, a multifaceted approach involving social and technical interventions is essential [25]. It follows that we need the willingness of stakeholders (such as owners of private ranches in priority areas for conservation), academics, the local communities, and policymakers to first recognize the potential problems and conflicts and then collaboratively discuss them and take action [22]. In the next sections, we present some of the most important collaborative efforts we are implementing in Argentina to mitigate the effects of human–primate conflicts.

### 5.1. A National Primate Conservation Plan

The National Primate Conservation Plan of Argentina, developed in 2019, by a team of primatologists and regional and national governmental authorities, was approved by the national government in 2021 (Res. 430/21, Ministerio de Ambiente y Desarrollo Sostenible- https://www.argentina.gob.ar/normativa/nacional/resoluci%C3%B3n-430-2021-358740/texto, accessed on 20 October 2023). The main goals of the plan were focused on (1) protecting and maintaining native forests and reducing habitat loss in the primate distribution areas, (2) maintaining and increasing connectivity between primate populations across different regions, (3) restoring and/or enriching degraded forests that are primate habitats, (4) implementing the effective management of native forests to avoid the degradation of the primates´ habitats, (5) assessing and reducing the impact of yellow fever on populations, (6) reducing the extraction of wild primates from their habitats and its associated effects, and (7) implementing environmental education programs focused on the conservation of primates. Most of the initiatives described below are the result of the implementation of concrete objectives derived from the main goals of the plan.

### 5.2. Priority Areas for Conservation

The effective creation and management of areas of importance for the conservation of primates could help mitigate some of the conflicts. A recent study estimated that only 7.2% (19,500 km^2^) of the area inhabited by primates in Argentina is under protection. To identify new priority areas, the authors performed a spatial conservation prioritization analysis based on primate habitat quality and connectivity to identify potential areas of importance at national and ecoregional levels. The top 1% priority areas were in the Atlantic Forest of Misiones province, where capuchins (*S. nigritus*) and two species of howlers (*A. guariba, A. caraya*) are distributed, and in the humid portion of eastern Chaco and Formosa provinces, where owl monkeys (*A. azarae*) and howlers (*A. caraya*) are present. The top 5% areas also included portions of the Yungas, where capuchins (*S. cay*) is the only primate present [148].

While protected areas continue to play a crucial role in species conservation efforts, it is important to acknowledge the limitations, given that not all primate habitats can be strictly protected. It is then important to implement community-based conservation practices and ethnoprimatology for the effective management and preservation of primate populations [25,28,35]

### 5.3. Population Management for the Conservation of the Brown Howler Monkey

Another initiative is to evaluate and reduce the impacts of yellow fever on the population of brown howler monkeys. The brown howler, *A. guariba*, was listed both in 2018 and 2022 as one of the 25 most threatened primate species worldwide [149]. It is the species most affected by yellow fever. There are only six confirmed localities for the species in the province of Misiones, and a population estimate of 20–50 adult individuals remaining in the country. Since the situation is so critical, researchers recently conducted a workshop to develop the most appropriate management plan for the species [150]. The agreed plan includes informed debates about the risks and benefits of reintroduction, rescue, population reinforcement, and ex-situ programs. Some of the measures also aim to recover the value that the brown howler monkey has for local communities: howlers are seed dispersers [151], sentinels of diseases like yellow fever (i.e., they show high mortality when infected by the virus, which allows for the establishment of control and prevention measures early on when death monkeys are found), and their howls are characteristic of the forests of Misiones. Reinforcing the value of primate species for local communities has been identified as a powerful mechanism to mitigate the consequences and negative effects of human–primate conflict [152].

### 5.4. Provincial Legislation: Natural Monuments

As noted above, only a small portion of the habitat used by primates is currently under protection. It is then imperative that we consider measures that are appropriate for protected areas that neighbor primate ranges; these could be on private or state land. One mechanism by which this can be accomplished is through the creation of Natural Province Monuments. It involves assigning primate species with a legally protected status; the status of becoming a “provincial monument”. This status directly mitigates and terminates conflicts since it provides a legal context that determines the animal, whether considered a “problem” for humans or not, can only be repelled, removed, or tolerated, but not killed.

There have been great advances in the last decade for declaring new natural monuments. For instance, owl monkeys (*A. azarae*) were considered natural monuments in the province of Formosa (Law 1.582/12, 2012) [153] in 2012 [154,155]; since then, howler monkeys (*A. caraya*) have been declared as such in the provinces of Corrientes (Law 6.590/21, 2021) [156] and Misiones (Law XVI N° 154, 2022) [157] in 2021 and 2022, respectively. Ongoing efforts are seeking to include capuchin monkeys (*S. nigritus*) to the list of monkeys declared natural monuments in Misiones Province. Other provinces with primates such as Chaco or Salta have not yet initiated this process.

### 5.5. Citizen Science and Environmental Education in Argentina

Conservation and environmental education can be useful in mitigating the effects of human–primate conflicts and in creating spaces for productive research implementation [158,159,160]. While educational and awareness initiatives may not provide a specific or technical conflict resolution, they may still enhance comprehension of primate behavior, leading to decreased harm in various situations (from interaction with primates at tourist sites to urban setting encounters). For instance, if people are better informed on how to behave when encountering primates, or if researchers have a deeper understanding of the local communities, these programs can reduce situations of conflict. For example, the representation and signification of primates (*A. pigra* and *Ateles geoffroyi vellerosus*) of the Q’eqchi’ Mayan community of Guatemala has led to positive attitudes and actions that not only limit monkey hunting but also favor the conservation of their habitat. They make agricultural decisions considering primate habitats, refrain from cutting down these forests, and plant native fruit trees to provide food for the primates. These participatory conservation experiences can serve to promote a more holistic, inclusive, and effective approach to conserving endangered primates and landscapes in Latin America [161]. Nonetheless, it is worth noting that many human–primate conflicts are derived from forest destruction and habitat loss, which are a result of global consumption and expansion. While many educational programs have focused on “educating” humans who live in communities near or in primate habitats, we need approaches to conservation education from broader angles that also include local stakeholders, policymakers, government officials, and the humans living in industrialized nations who are major consumers of the items that lead to forest loss [162].

There are various conservation and environmental educational programs in Argentina focused on primates. In the province of Corrientes, researchers from the Corrientes Biological Station (EBCO) have been actively working with a group of high school students (i.e., Club de Ciencias Arquimedes—Inst. PIO XI) to transform the perception of howler monkeys held by locals. The work led to the creation of the group Guardianes del Carayá [163], which, among other activities, resulted in the finding of a location to install bridges to facilitate the movement of howler monkeys across gaps in a discontinued canopy. The students worked to build awareness within their community, obtained the necessary permits, designed the bridges, and installed three of them [76]. Unfortunately, the bridges are not yet equipped with camera traps or other automatic devices to register the use of the bridge by the monkeys and/or other animals. As a result, direct evidence of their effectiveness in mitigating landscape fragmentation is currently unavailable. Nonetheless, a similar approach with mantled howler monkeys (A. palliata palliata) in Costa Rica demonstrated success. After bridge implementation, and throughout 2015–2016, results showed that the number of monkey groups increased, their home ranges expanded, the population grew, and fatalities decreased [104].

Additionally, participatory actions in Corrientes were conducted in collaboration with the Nordeste National University´s initiative UNNE+SALUD, in 2019. The project, building on research with howler monkeys (*A. caraya*) and foxes (*Cerdocyonthous, Lycalopex gymnocercus*) in urban–rural interface zones at the Corrientes Biological Station (EBCO), proposed strategies for early disease detection through citizen involvement [164,165,166]. This holistic approach was in line with what is proposed by the One Health framework in primatology, which promotes diverse and transdisciplinary practices for disease risk analyses, disease prevention, health monitoring and disease surveillance, clinical interventions, and community engagement [167].

**Figure 7:**
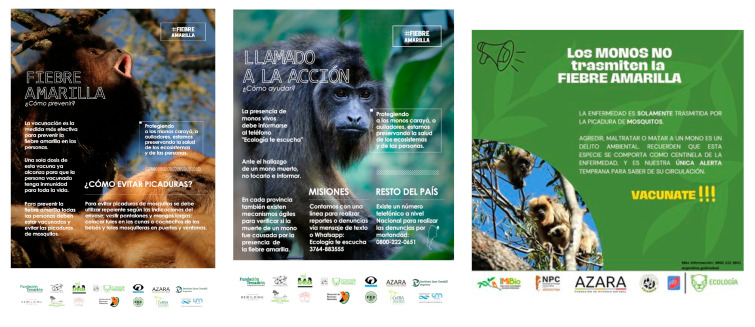
Posters of the Yellow Fever Campaign that were publicly shared on social media during 2021–2023.

More broadly, at a national level, the work of the Argentinean Jane Goodall Institute (www.janegoodall.org.ar, accessed on 20 October 2023) has actively engaged in various conservation and education efforts since 2011. As part of the work toward preserving native habitats and species, the institute implements educational programs in schools and communities, raising awareness about the significance of biodiversity and primates. In the last several years, with the support of other organizations, they led the public Yellow Fever Campaign (Campaña de Fiebre Amarilla), which was extensively shared on social media (Figure 7).

Lastly, it is worth explaining that Argentina passed a law in 2021 that establishes the implementation of integral environmental education in the country (Law 27.621/21) [168]. The law provides the framework for environmental education, emphasizing the importance of integrating, at all levels, environmental knowledge and values into formal and non-formal education systems. It aims to promote sustainable development, environmental conservation, and responsible citizenship by fostering a deeper understanding of environmental issues and encouraging active participation in environmental protection efforts. It underscores the collaboration between government agencies, educational institutions, and civil society to develop and implement effective environmental education programs throughout Argentina.

### 5.6. Primate Research Groups in Argentina

There are several research groups focused on the study and conservation of primates in Argentina. While their research foci are different, collectively their studies have provided a solid theoretical framework from which to assess the consequences of conflict between humans and primates. All research groups come together in the Argentinean Primatological Association (Asociación de Primatología Argentina, APRIMA, www.aprima.com.ar, accessed on 20 October 2023). APRIMA was founded in 2009 to provide guidelines for the execution of effective research and conservation of the primates of Argentina.

**Proyecto Mirikiná/The Owl Monkey Project** (www.owlmonkeyproject.com, accessed on 20 October 2023): Established in the province of Formosa in 1996, the Owl Monkey Project (OMP) of Fundación ECO has emphasized the study of *Aotus* and the flora and fauna of the Humid Gran Chaco. Employing diverse biological and ecological methods, the team has taken a multidisciplinary approach and combined studies of population biology, demography, behavior, genetics, endocrinology, and ecology [59,155,169,170,171,172,173]. The OMP was instrumental in providing the information required to update the IUCN status of *A. azarae* [174], as well as new information on their ecology and behavior for the recent categorization of primate conservation in the country [175] and in declaring them a Natural Monument in the province of Formosa.

**Estación Biológica Corrientes** (EBCo, www.biologicaestacion.wixsite.com/ebco, accessed on 20 October 2023): EBCo is located in the province of Corrientes, and affiliated with the Centro de Ecologia Alicada del Litoral—National Argetinean Research Council (Consejo Nacional de Investigaciones Científicas y Técnicas, CONICET and Universidad Nacional del Nordeste) [176]. This institution includes a multidisciplinary group of researchers, undergraduate and graduate students, and technical personnel who focus their research on the ecology, behavior, demography, genetics, and impact of infectious diseases on howler and other wild animal populations in areas of contact with humans and domestic animals, as well as in pristine areas. The EBCO has become a national leader in the implementation of environmental and conservation education in the country through several decades of continuous work with local schools and universities.

**Neotropical Primates Conservation-Argentina** (NPC, www.neoprimate.org/es/npc-argentina-es/, accessed on 20 October 2023), Argentina: Founded in 2021 (the main research activities of NPC include studies on ecology, genetic structure, infectious disease dynamics, and the biogeography of the two howler monkey species native to Argentina, the black-and-gold howler monkey (*A. caraya*) and the brown howler monkey (*A. guariba*), both of which are important indicator species for disease outbreaks. They also conduct research on capuchin monkeys (*S. nigritus*), principally on how habitat fragmentation and anthropogenic disturbance affect their genetics, ecology, and health. Several years of continuous fieldwork with these species, with different physical characteristics (isolated, continuous, urban, or rural, for example), have been fundamental in understanding the impacts of habitat alterations on primate population dynamics, particularly those effects caused by natural and anthropogenic dispersal barriers and habitat loss in Argentina. Through international collaborations, the NPC Argentina team uses sniffer dogs in areas of low primate densities to find feces from which they obtain genetic material for analysis to detect diseases and parasites, and monitor genetic profiles. They are currently implementing programs of citizen science, which include working with veterinarians, volunteers, students, and local communities, who can provide important information to evaluate the genetic distribution of primates and identify risk areas for disease transmission. To this end, they have developed a genotype database (https://doi.org/10.5281/zenodo.3378896) that can be used to cluster genetically distinct populations of *A. caraya*.

**Proyecto Caí** (www.ceiba.org.ar, accessed on 20 October 2023): The Proyecto Caí has been studying the behavior and ecology of *S. nigritus* in the Iguazú National Park since 1991. The project includes Argentinean researchers from the Institute of Subtropical Biology (IBS), and the Civil Association of the Atlantic Forest Research Center (CeIBA), as well as scientists from institutions in different countries around the world.

## 6. Conclusions

In line with the most common conservation threats faced by primates worldwide, across their southernmost distribution, monkeys in Argentina are challenged with the effects of habitat loss and disease epidemic [5,6]. These threats further generate contexts of conflict between humans and primates with negative consequences for all actors involved. In Argentina, the context of conflict derived from habitat loss and landscape modification manifests in various forms. Anthropogenically induced fires, used uncontrollably for grassland management in private ranches, have had catastrophic consequences for howler monkeys, as evidenced by incidents at research sites like the Estación Biológica de Corrientes. Urbanization further presents a new dimension of conflict for primates. As human populations expand, primates in Argentina increasingly encounter threats such as electrocutions, roadkills, and retaliatory killing due to crop foraging in urban and suburban environments. Additional contexts of conflict in the country are the ones induced by human misconceptions about primates´ biology and behavioral ecology. For instance, during the yellow fever outbreaks, besides the deaths of the monkeys caused by the virus, misconceptions and unfounded fears led humans in rural areas to kill the monkeys when they were encountered. Food provisioning, particularly in tourist areas like National Parks, and detrimental practices in rescue centers, such as close contact with humans or unsupervised relocations, are further posing challenges to monkeys in Argentina.

Our manuscript showed that in Argentina, visible or direct contexts of conflict are more frequent with primate species found in urban or tourist areas (i.e., *A. caraya* and *S. nigritus*). However, the scarcity of data for other species is likely due to their presence primarily in protected or more inaccessible areas, rather than due to the lack of conflicts. For example, the extremely limited number of *A. guariba* individuals in the country, as well as their restricted habitat in Misiones, contributes to the absence of data on roadkill, or pet trading. In other words, we propose that conflicts between humans and primates in the country are probabilistic and mediated by the number of individuals of each species and their use of space.

These complex issues underscore the need for conservation efforts, comprehensive education, and research-driven interventions, to protect primate populations in the country and to mitigate the negative effects of conflict. It follows that collaboration among academics, local communities, stakeholders, policymakers, and government authorities is essential to recognize and tackle these challenges effectively in Argentina. The recent elaboration and governmental approval of a National Primate Conservation Plan (Res. 430/21, Ministerio de Ambiente y Desarrollo Sostenible) founded the bases and directed the actions toward a multifaceted approach that included the identification of priority conservation areas, the collaborative management plan of the most endangered species in the country (*A. guariba*), the designation of legally protected status to owl monkeys and howler monkeys in various provinces, and the effective implementation of citizen science and environmental education. These comprehensive strategies aim to safeguard primate populations and mitigate the adverse impacts of human–primate conflicts in Argentina.

## Figures and Tables

**Table 1:** List of Argentinean primate species, the different types of habitats they inhabit, the categorization of extinction risk levels by the IUCN and the Sociedad Argentina de Especialistas en Mamíferos (SAREM), and the conservation threats identified in the country.

Species	Habitat/Ecoregion	IUCN Categorization (2023)	SAREM Categorization (2019)	Conservation Threats in Argentina
*Alouatta caraya*	Dry and Humid Chaco Forests and Paranaense Rainforest	Near Threatened	Vulnerable	Habitat Loss and Degradation, Diseases, Illegal pet trade
*Alouatta guariba clamitans*	Paranaense Rainforest	Vulnerable	Critically Endangered	Habitat Loss and Degradation, Diseases, Roadkills
*Aotus azarae*	Dry and Humid Chaco Forests	Least Concern	Vulnerable	Habitat Loss and Degradation, Illegal pet trade
*Sapajus nigritus cucullatus*	Paranaense Rainforest	Near Threatened	Vulnerable	Habitat Loss and Degradation, Illegal pet trade, Tourism
*Sapajus cay*	Yungas	Vulnerable	Vulnerable	Habitat Loss and Degradation, Illegal pet trade

**Table 2:** The two main drivers of the different contexts of conflict between humans and primates in Argentina. The last column indicates approaches implemented in the country to mitigate the negative consequences of conflicts.

Drivers	Context of Conflicts	Approaches to Mitigate Conflict
Habitat loss	(A) Anthropogenically induced Fires	Protected areas
	(B) Urbanized monkeys	Natural Provincial Monuments, Educational Programs
	(C) Electrocutions	Canopy bridges
	(D) Roadkills	Canopy bridges
	(E) Crop foraging	Protected areas, Educational Programs
Human misconceptions	(A) Infectious diseases	Protected areas, Educational Programs
	(B) Food provisioning	Educational Programs
	(C) Reintroductions and relocations	Protected Areas, Research
	(D) Illegal Pet trade	Natural Provincial Monuments, Educational Programs

## Data Availability

Not applicable.
